# Prevalence and predictors of condom use among people who inject drugs in Georgia

**DOI:** 10.1186/s12954-025-01171-6

**Published:** 2025-02-20

**Authors:** Maia Kajaia, Maia Butsashvili, Jack A. DeHovitz, George Kamkamidze, Lasha Gulbiani, Tinatin Abzianidze, Mamuka Djibuti

**Affiliations:** 1 Ivane Javakhishvili Tbilisi State University, 1 Chavchavadze Ave, Tbilisi, 0179 Georgia; 2 Health Research Union (HRU), 8 Nutsubidze Street, Tbilisi, 0177 Georgia; 3 SUNY Downstate Health Sciences University, 450 Clarkson Ave, Brooklyn, NY 11203 USA; 4 Partnership Research Action Health (PRAH), 3 B. Zghenti Street, Tbilisi, Georgia

**Keywords:** Condom, PWID, HIV, Unsafe, Behavior, Consistent

## Abstract

**Background:**

People who inject drugs (PWID) are more likely to engage in unsafe sexual behavior placing them at high risk of acquiring HIV and other STIs. This study aims to assess the prevalence and predictors of inconsistent condom use with casual and/or paid sexual partners among PWID in Georgia.

**Methods:**

Integrated Bio-Behavioral Surveillance Survey was conducted among PWID in seven major cities of Georgia. Study design was cross-sectional with respondent-driven sampling (RDS) methodology. Data collection was carried out through individual face-to-face interviews. Of the 2005 PWID who participated in the study, we analyzed a subsample of 619 (30.9%) who reported having casual and/or paid sexual partners during the last 12 months and described prevalence and predictors of consistent condom use.

**Results:**

Consistent condom use during casual and/or paid sex in past 12 months was reported by 49.4% of respondents. The likelihood of consistent use with casual and/or paid sexual partners was statistically significantly associated with residence, family income, drug use frequency, drug dependence and HIV risk self-perceptions. In multivariable analysis independent predictors of always using condom at casual/paid sex during the last 12 months were place of residence (aOR = 6.4; 95% CI: 3.2–12.7), family income (aOR = 2.1; 95% CI:1.3–3.5) and drug use frequency (aOR = 0.6; 95% CI: 0.4–0.9).

**Conclusion:**

The study revealed low prevalence of consistent condom use with casual and/or paid sexual partners among PWID in Georgia. Integration of safe sex educational interventions in harm reduction services may improve the rates of condom use among PWID and should focus PWID with lower socio-economic status and residing outside capital city.

## Introduction

Georgia is considered a low HIV prevalence country (estimated 0.4% HIV prevalence in the adult population) with cases of HIV infection concentrated among key affected populations including people who inject drugs (PWID) [1]. The country has one of the highest rates of injection drug use in the world [2]. National prevalence estimates for injection drug use are 2.23% in 18–64 years old population, and 1.39% in general population [3]. According to Georgian AIDS and Clinical Immunology Research Center 32.6% of HIV cases are among PWID [4].

Although parenteral exposure resulting from unsafe injection behavior is the main cause of HIV infection in people who inject drugs (PWID) [5, 6], a significant proportion of HIV infection in this population is sexually transmitted [7, 8]. In recent years, sexual transmission has become the predominant mode of HIV spread in Georgia. Initially, injection drug use was the primary route; however, since 2012, sexual transmission has surpassed it. In 2024, heterosexual transmission accounted for 52.4% of all cases, and heterosexual transmission among MSM constituted 13.7% of total cases identified that year [4]. PWID are more likely to engage in unsafe sexual behavior, placing them at high risk of acquiring HIV and other sexually transmitted infections (STIs), as well as subsequent transmission to non-drug-using sexual partners [9–13]. Correct and consistent use of condoms is an effective measure of preventing transmission of STIs including HIV [14–16]. While, condom use is one of the main interventions to prevent HIV infection for key affected populations and general public [17–19], interventions among PWID are mainly focused on reduction of injection risk behaviors [20, 21]. Unsafe injection practices can be significantly reduced with such interventions, but unsafe sexual behavior among PWID is difficult to modify [22, 23] and multiple studies show high rates of unprotected sex among PWID [10, 24–27]. Although biomedical prevention strategies such as pre-exposure prophylaxis (PrEP) and treatment as prevention (TasP) have been shown to be highly effective in preventing HIV transmission [28, 29], these approaches are not widely utilized among PWID in Georgia. PrEP is not commonly accessed by PWID, and existing data suggest that ART coverage and viral suppression rates among this population remain low, limiting the protective effects of TasP [30, 31]. Given this context, condom use remains the primary and most accessible method of HIV and STI prevention for PWID in Georgia. Therefore, addressing barriers to consistent condom use remains an urgent public health priority.

PWID can access HIV preventive services at Georgian Harm Reduction Network (GHRN) centers, which provide free services such as screening for HIV, hepatitis B and C, syphilis, and tuberculosis, as well as sterile paraphernalia (various size syringes, needles, butterfly needles, alcohol pads, tourniquets, Naloxone, injection solutions), and sexual health supplies including condoms and lubricants. Additionally, educational materials (flyers, brochures, booklets) on HIV/AIDS and informational sessions are available. Despite the availability of harm reduction services, there are gaps in addressing sexual risk behaviors among PWID. While condoms and sexual health information are provided, data on their coverage and usage trends remain limited. Moreover, sexual risk behavior reduction counseling is not part of the existing harm reduction services, meaning that while condoms are distributed, there is no structured guidance on their consistent and effective use.

Given that PWID are at higher risk of HIV and other STIs compared to the general population they represent a vulnerable population who need specific approach addressing not only drug-related but also sexual risk behaviors. This study aims to assess the prevalence and predictors of inconsistent condom use with casual and/or paid sexual partners among PWID in Georgia.

## Methods

An Integrated Bio-Behavioral Surveillance Survey (IBSS) was conducted among PWID in seven major cities of Georgia: Tbilisi (capital city), Gori, Rustavi, Telavi, Batumi, Zugdidi, and Kutaisi. Study design was cross-sectional. Study participants were recruited by respondent-driven sampling (RDS) utilizing recruitment of research participants by other participants. The RDS method is based on social network theory and includes non-probability “snowball sampling” with mathematical modeling, which allows weighing the sample [32]. While respondent-driven sampling (RDS) was initially developed to generate population-based estimates, studies have shown that it often fails to achieve this goal, leading to biased estimates. Factors such as the influence of the initial seed sample, network homophily, and preferential recruitment contribute to these biases, limiting the accuracy of RDS-derived estimates. Despite these methodological challenges, RDS remains widely used worldwide as it provides a practical means to include hard-to-reach populations in research, particularly in epidemiologic studies on key populations at high risk for HIV and other infectious diseases [33–35].

The recruitment of study participants included a double incentive system: a primary reward for participating in the study and a secondary reward for recruiting other PWID into the study. The primary reward was 20 GEL (approximately 7 USD), and the secondary reward was 10 GEL (approximately 3.5 USD) for the inclusion of each new respondent in the study. Study participants were selected according to the following inclusion criteria: age ≥ 18 years, drug injection practice at least once in the 30 days prior to the survey, residence in the selected cities where the survey was conducted, willingness and ability to give informed consent for study participation. The assessment of potential study participants for being PWID was done by informal interview addressing drug prices, slang names, preparation, and injection techniques. In addition, we assessed injecting drug use through visual inspection of objective signs, such as track marks, skin changes, or other physical indicators commonly associated with injection. Each eligible potential study participant was informed about the purpose, objectives, methods, procedures, risks, and benefits of the study. All individuals who agreed to participate in the study signed an informed consent form and then were enrolled in the study. We started RDS sampling by purposive selection of “seeds”– initial study subjects representing target population. Besides the study inclusion and exclusion criteria, additional factors were considered during the selection of “seeds”. Namely, “seeds” should have access to different groups of PWID, which ensured a diversity of the sample. Different ages, social and geographical characteristics were also considered during the selection of “seeds”.

The study included behavioral and biomarker components. The behavioral component data collection was carried out through individual, face-to-face interviews. The survey tool was a structured questionnaire collecting the following information: socio-demographic characteristics, injection practices, sexual behavior, use of HIV-preventive programs, and social factors related to drug use. The biomarker component of the study included testing of blood samples for HIV infection, hepatitis B and hepatitis C (the results of this component will be reported elsewhere).

Before initiation of field work, the study protocol and instruments were reviewed and approved by Institutional Review Board of Health Research Union (IRB00009520; IORG005619).

In total 2005 PWID participated in the study. In this paper we analysed a subsample of 619 PWID who reported having casual and/or paid sexual partners during the last 12 months and described prevalence and predictors of consistent condom use. “Casual sexual partner” was defined as a sexual partner who is not a regular partner and with whom a sexual relationship is established without financial compensation. “Paid sexual partner” was defined as a sexual partner with whom a sexual relationship is established in exchange for material remuneration (pays the partner or receives remuneration from the partner). “Consistent condom use” was defined as self-reported “always using condom” with casual and/or paid sexual partners during the last 12 months. Descriptive statistical methods were used to characterize socio-demographics, sexual behavior and condom use of study population. In bivariate analysis study variables were compared between different study groups (participants who consistently used condoms and those who did not) using chi-square test for categorized data. Logistic regression model was used for multivariable analysis to identify independent predictors of consistent condom use. To construct the model, we employed stepwise regression. Initially, we assessed each variable for its unique contribution to the model. Variables that did not contribute were removed but were later reintroduced if they demonstrated a greater ability to explain variance in the dependent variable than during their initial inclusion. The criteria for including or excluding variables were determined based on p-value. We report unadjusted and adjusted odd ratios (aORs) with 95% Confidence Intervals (CIs). The p-value of < 0.05 was considered significant.

## Results

As noted above 619 PWID (30.7%) reported having casual and/or paid sex during the last 12 months. Almost all participants (*n* = 612, 98.9%) were males. The age of the respondents ranged from 18 to 67 years (median age 39 years). The vast majority of the study participants were ethnically Georgian (*n* = 605, 97.7%). 30.7% (*n* = 190) held a university degree. 33.6% (*n* = 208) of the surveyed PWID were married and almost two thirds (*n* = 384, 62.0%) were unemployed. Most of the respondents (*n* = 491, 79.4%) lived with their spouse/partner, parents, or relatives. Most of the study participants’ family income (*n* = 210, 55.9%) was less than 700 GEL (270 USD) per month.

Table 1 describes sexual behavior and condom use with casual and paid sexual partners. Most of the respondents (*n* = 491, 79.9%) had their first sexual intercourse before the age of 18. The median age of sexual debut was 16 years. The majority of surveyed individuals (*n* = 396, 64.9%) had three or more sexual partners during the last 12 months. 329 (54.5%) of the study subjects reported using a condom during the last sexual intercourse with any sexual partner and 460 (74.3%) with casual and/or paid sex partner. Nearly two thirds of the interviewed PWID (*n* = 375, 61.8%) indicated that were under the influence of drugs during the last sexual contact. Most of the study subjects (*n* = 517, 83.5%) did not have any problems with obtaining condoms. Condom use during the last sexual intercourse was the result of a shared decision with the partner among 98 (46.4%) of PWID, in 68 (32.2%) - only the respondent’s decision, and in 29 (13.7%) - only the partner’s decision. The main reasons for not using a condom at last sexual contact were: “not considering necessary to use condoms” (*n* = 9, 42.9%) and “not liking to use condoms” (*n* = 5, 23.8%). Consistent condom use with casual and/or paid sex during the last 12 months was reported by only 306 (49.4%) of the respondents.

By bivariate analysis the likelihood of consistent condom use with casual and/or paid sexual partner was statistically significantly associated with residence, family income, drug use frequency, drug dependance and HIV risk self-perceptions. The probability of always using condom during casual/paid sex was higher among the respondents residing in Tbilisi (73.2%) compared to those living in other regional cities (43.3%) (OR = 3.5; CI: 2.3–5.5). PWID with family income ≥ 500 GEL (approximately 200 USD) were more likely to use condoms consistently with casual/paid sexual partners than those with lower income (57.3% vs. 41.5%, OR = 1.8; 95% CI:1.2–2.9). A lower proportion of the respondents who injected drugs frequently (once a week or more) always used condom at casual/paid sex compared to more rare injectors (once or several times per month) (46.7% vs. 56.5%; OR = 0.6; 95% CI:0.4–0.9). PWID who didn’t perceive themselves as drug dependent (56.0%) were more likely to report consistent condom use at casual/paid sex, than those who did (45.8%) (OR = 1.5; 95% CI:1.1–2.1). HIV risk self-perception was also associated with consistent condom use, as higher percentage of the respondents who thought that they were under the risk of contracting HIV were always using condom with casual/paid sexual partners, compared to those who didn’t consider themselves at risk of HIV (50.8% vs. 35.7%; OR = 1.8; 95% CI: 1.1–3.2) (Table 2).

In multivariable analysis independent predictors of always using condom at casual/paid sex during the last 12 months were place of residence (aOR = 6.4; 95% CI: 3.2–12.7), family income (aOR = 2.1; 95% CI:1.3–3.5) and drug use frequency (aOR = 0.6; 95% CI: 0.4–0.9) (Table 2).

## Discussion

This study revealed high levels of unsafe sexual behavior among PWID in Georgia. The findings indicate that substantially higher proportion of the interviewed PWID had multiple sex partners (64.9% had ≥ 3 sexual partners) compared to other studies conducted in different countries where this indicator varied between 14 and 47% [36–40]. We also found a high prevalence of unprotected last sexual intercourse with any type of sexual partner among PWID, and this finding is consistent with other studies [41–44].

Consistent condom use during casual and/or paid sex was reported only by 49.4% of PWID. Other studies also showed the high rates of inconsistent condom use among PWID [24, 44–47]. Low rates of consistent condom use among PWID can be a consequence of sex under influence of drugs. Many PWID take drugs before sexual intercourse which can influence their decision and negotiation with partner on condom use. One important finding was that the frequency of drug use showed a positive association with inconsistent condom use. This finding is in line with previous studies suggesting that substance use leads to unsafe sexual behaviors [48–51]. Drug use might decrease the perception of unsafe behaviors and capacity to control these behaviors among PWID and thus could facilitate the engagement into unsafe sexual behaviors. It underlies the importance of enrollment and adherence to opioid agonist therapy (OAT) among PWID, as being on OAT, adhering to treatment and terminating injection drug use could lead to safer sexual practices and decreased risk of HIV and other STI transmission [39, 52].

We found that the study participants who had higher family income were more likely to use condoms constantly with casual and/or paid sexual partners. It seems that PWID with low family income cannot afford to buy condoms, thus it is very important among key populations to enhance programs that promote condom use not only through education but also increase access to condoms [53]. Our opinion is supported by a study conducted by Song YS et al. finding that taking condoms from clinic stocks was the best predictor of condom possession, which in turn was the best predictor of condom use among men enrolled in drug treatment programs [54].

Our study also showed that living in other cities was associated with higher odds of inconsistent condom use compared to living in Tbilisi (capital city). IBSS survey conducted in 2009 among PWID in Georgia showed similar association with place of residence regarding inconsistent condom use and “dual risk behavior” defined as both unsafe injecting behavior at last injection and not using condom at last casual and/or paid sex [55, 56]. This means that PWID from regional areas of the country are still more likely to practice unsafe sexual behaviors suggesting the need for additional behavioral health education about safe sex practices among PWID residing outside the capital city. Health education targeted at individual’s risk self-perception, behavioral and normative beliefs would likely influence sexual risk behaviors among PWID. In addition, there must be increased efforts to reduce drug use as previous studies have shown that behavioral health education in combination with OAT have positive impact on reduction of HIV related risk behaviors [57, 58].

While Prevalence Ratios (PRs) are often preferred in cross-sectional studies, we chose to report Odds Ratios (ORs) due to the robustness and flexibility of logistic regression in assessing associations between predictors and binary outcomes. ORs facilitate comparability with previous studies on condom use among PWID and provide stable estimates even when convergence issues arise in log-binomial regression. Although ORs may overestimate the strength of association when the outcome is common, they remain widely accepted in epidemiological research.

The study had some limitations. First, data was collected through face-to-face interviews, therefore may be subject to social desirability bias which is particularly problematic in studies involving sexual behavior, as respondents may not accurately answered some of the sensitive questions, either by underreporting stigmatized activities or by overreporting normative ones, if their actual behavior is considered socially unacceptable. Second, the findings rely on the study participants’ self-reported data which can be accompanied by recall bias, as the study participants may have had difficulties in recalling information about their sexual behavior in the past 12 months. Third, because of cross-sectional study design, it is not possible to make causal inferences. Fourth, the study sample consisted predominantly of male participants, which reflects the actual gender distribution of PWID in Georgia. Since drug use is overwhelmingly male dominated in the country, this study accurately represents the population rather than being a result of recruitment bias. However, this gender imbalance may influence the generalizability of findings, as risk behaviors and prevention needs could differ among female PWID.

## Conclusion

The study highlights low prevalence of consistent condom use with casual and/or paid sexual partners among PWID in Georgia. Integration of education about safe sexual practices into harm reduction services is an important component to decrease unsafe sexual practices and improve the rates of condom use among PWID in Georgia. Safe sex educational interventions should focus PWID with lower socio-economic status and residing outside capital city.

**Table 1:** Sexual behavior and condom use

Characteristics	Descriptive statistics		RDS estimates		
	N	%	%	95%CI	
				Lower	Upper
**How old were you when you had the first sexual intercourse?**					
< 18 years old	491	79.9	77.9	65.9	86.5
≥ 18 years old	112	18.1	18.8	11.0	30.1
Don’t know	16	2.5	3.2	1.6	6.4
**Median age of beginning sexual life (min-max)**	16 (13–24)				
**In total, with how many sexual partners have you had during the last 12 months?**					
1	43	7.0	6.3	3.8	10.1
2	126	20.7	21.4	17.4	25.9
≥ 3	396	64.9	64.6	58.4	70.3
Don’t know/ No response	45	7.4	7.8	4.9	12.0
**Did you use a condom during the last sexual intercourse?**					
Yes	329	54.5			
No	263	43.5			
Don’t know/No response	12	2			
**Did you use condom during the last casual and/or paid sexual intercourse?**					
Yes	460	74.3	74.8	68.1	77.4
No	159	25.7	25.2	31.9	22.6
**Were you or your sexual partner under the influence of drugs during the last sexual intercourse?**					
Yes, I was	375	61.8	63.5	55.6	70.7
Yes, my sexual partner was	3	0.5	0.5	0.2	1.7
Yes, both me and my sexual partner were	32	5.3	5.5	3.5	8.7
No	153	25.2	22.4	15.8	30.7
Don’t know/No response	44	7.2	7.9	3.9	14.8
**Have you had any problem(s) obtaining condoms during the last month?**					
Yes	21	3.4	3.3	2.0	5.5
No	517	83.5	83.9	77.0	89.0
Don’t know	5	0.8	0.6	0.2	1.8
No response	76	12.3	12.1	7.4	19.1
**Whose decision was to use condom during the last sex?**					
My decision	68	32.2	27.7	16.1	43.4
Partner’s decision	29	13.7	15.7	10.3	23.3
Shared decision	98	46.4	47.4	39.3	55.6
Don’t know	16	7.6	9.1	4.6	17.1
**Why you didn’t use condom during the last sex?**					
The partner refused	1	4.8	1.6	0.1	13.5
Don’t like it	5	23.8	19.4	5.9	48.1
Don’t think it was necessary	9	42.9	48.8	25.4	72.7
Didn’t think of that	4	19	26.5	9.0	56.8
Other	1	4.8	3.4	0.4	22.3
**Frequency of condom use with casual and/or paid sex partner(s) in last 12 months**					
Always	306	49.4	48.0	38.7	57.5
Not always	313	50.6	51.9	42.4	61.3

**Table 2:** Predictors of consistent condom use with casual and/or paid sexual partners

Characteristics	Consistent condom use with casual and/or paid sex partners		OR; 95% CI	*P* value	aOR; a95%CI	a*P* value
	*N*	%				
**Age**						
≤ 30 years	64	83.3	1.1 (0.7–1.6)	0.3		
> 30 years	242	48.9				
**Marital status**						
Married	103	49.5	1.0 (0.7–1.4)	0.9		
Other	203	49.4				
**Level of education**						
High school/Vocational college	169	46.7	1.3 (0.9–1.8)	0.9		
University	136	53.5				
**Residence**						
Tbilisi	93	73.2	3.5 (2.3–5.5)	< 0.0001	6.4 (3.2–12.7)	< 0.0001
Other	213	43.3				
**Family Income**						
< 500 GEL	81	41.5	1.8 (1.2–2.9)	0.02	2.1 (1.3–3.5)	0.002
≥ 500 GEL	86	57.3				
**Drug use frequency**						
Once or several times a month	121	56.5	0.6 (0.4–0.9)	0.02	0.6 (0.3-1.0)	0.07
Once a week or more	179	46.7				
**Alcohol consumption frequency**						
Never/Rarely	192	50.7	0.8 (0.6–1.2)	0.4		
Once a week or more	114	47.5				
**Drug dependence self-perception**						
Yes	202	45.8	1.5 (1.1–2.1)	0.02	1.5 (0.9–2.7)	0.9
No	89	56.0				
**HIV risk self-perception**						
Yes	280	50.8	1.8 (1.1–3.2)	0.03	2.2 (0.8–6.1)	0.1
No	20	35.7				
**Used preventive programs in last 1 year**						
Yes	168	56.4	1.3 (0.9–1.9)	0.9		
No	91	48.7				

## Data Availability

The data that support the findings of this study are not openly available due to reasons of sensitivity and are available from the corresponding author upon reasonable request. Data are located in controlled access data storage at Health Research Union, Tbilisi, Georgia.
